# Effects of methylprednisolone on blood-brain barrier and cerebral inflammation in cardiac surgery—a randomized trial

**DOI:** 10.1186/s12974-018-1318-y

**Published:** 2018-09-27

**Authors:** Mattias Danielson, Björn Reinsfelt, Anne Westerlind, Henrik Zetterberg, Kaj Blennow, Sven-Erik Ricksten

**Affiliations:** 1 Department of Anesthesiology and Intensive Care Medicine, Sahlgrenska University Hospital, University of Gothenburg, SE-413 45 Gothenburg, Sweden; 2 Deparment of Anesthesiology and Intensive Care Medicine, Sahlgrenska University Hospital, Sahlgrenska Academy, University of Gothenburg, SE-41345 Gothenburg, Sweden

**Keywords:** Aortic valve replacement, Cardiopulmonary bypass, Cerebrospinal fluid, Neuroinflammatory response, Methylprednisolone

## Abstract

**Background:**

Cognitive dysfunction is a frequent complication to open-heart surgery. Cerebral inflammation caused by blood-brain barrier (BBB) dysfunction due to a systemic inflammatory response is considered a possible etiology. The effects of the glucocorticoid, methylprednisolone, on cerebrospinal fluid (CSF) markers of BBB function, neuroinflammation, and brain injury in patients undergoing cardiac surgery with cardiopulmonary bypass were studied.

**Methods:**

In this prospective, randomized, blinded study, 30 patients scheduled for elective surgical aortic valve replacement were randomized to methylprednisolone 15 mg/kg (*n* = 15) or placebo (*n* = 15) as a bolus dose administered after induction of anesthesia. CSF and blood samples were obtained the day before and 24 h after surgery for assessment of systemic and brain inflammation (interleukin-6, interleukin-8, tumor necrosis factor-alpha), axonal injury (total-tau, neurofilament light chain protein), neuronal injury (neuron-specific enolase), astroglial injury (S-100B, glial fibrillary acidic protein), and the BBB integrity (CSF/serum albumin ratio).

**Results:**

In the control group, there was a 54-fold and 17-fold increase in serum interleukin-6 and interleukin-8, respectively. This systemic activation of the inflammatory cytokines was clearly attenuated by methylprednisolone (*p* < 0.001). The increase of the CSF levels of the astroglial markers was not affected. A postoperative BBB dysfunction was seen in both groups as the CSF/serum albumin ratio increased from 6.4 ± 8.0 to 8.0 in the placebo group (*p* < 0.01) and from 5.6 ± 2.3 to 7.2 in the methylprednisolone group (*p* < 0.01) with no difference between groups (*p* = 0.98). In the CSF, methylprednisolone attenuated the interleukin-6 release (*p* < 0.001), which could be explained by the fall in systemic interleukin-6, and the serum to CSF gradient of IL-6 seen both at baseline and after surgery. In the CSF, methylprednisolone enhanced the interleukin-8 release (*p* < 0.001) but did not affect postoperative changes in CSF levels of tumor necrosis factor alpha. Serum levels of S-100B and neuron-specific enolase increased in both groups with no difference between groups. CSF levels of total tau, neurofilament light chain protein, and neuron-specific enolase were not affected in any of the groups.

**Conclusions:**

Preventive treatment with high-dose methylprednisolone attenuated the systemic inflammatory response to open-heart surgery with cardiopulmonary bypass, but did not prevent or attenuate the increase in BBB permeability or the neuroinflammatory response.

**Trial registration:**

Clinical Trials, Identifier: NCT01755338, registered 24 December 2012

**Electronic supplementary material:**

The online version of this article (10.1186/s12974-018-1318-y) contains supplementary material, which is available to authorized users.

## Background

Neurological complications to open-heart surgery remain a common cause of postoperative morbidity [1, 2]. They span from the, often reversible, condition of postoperative cognitive dysfunction (POCD) and delirium to manifest stroke. The, possibly multifactorial, etiology remains unclear but arteriosclerosis, cerebral microembolization originating from cardiopulmonary bypass (CPB), cerebral inflammation, blood-brain barrier (BBB) disruption, and cerebral hypoperfusion/hypooxygenation have been suggested as possible causes [3].

Experimental studies have suggested that surgical trauma induces a systemic inflammatory response causing a disruption of the blood-brain, neuroinflammation, and cognitive dysfunction [4, 5]. Cardiac surgery is associated with a profound systemic inflammatory response due to the surgical trauma and the interaction between blood and the artificial surfaces of the CPB circuit [6]. Previous studies have demonstrated the presence of brain edema early after cardiac surgery with CPB [7, 8], which could to some degree be explained by a BBB dysfunction [3, 9].

In a previous study, we studied the release of cerebrospinal fluid markers of inflammation, neuronal and glial cell injuries, and BBB function after cardiac surgery with CPB [10]. A pronounced cerebral inflammatory response was found together with glial cell injury and BBB dysfunction, without biochemical signs of neuronal injury.

High doses of corticosteroids can be used to suppress the postoperative systemic inflammatory response syndrome in cardiac surgery [11]. Methylprednisolone is a synthetic glucocorticoid, which has been shown to suppress the release of systemic pro-inflammatory cytokines in patients exposed to cardiac surgery with CPB [11]. If the neuroinflammatory response to cardiac surgery with CPB is triggered by a systemic inflammation, an inhibition of this systemic inflammation by high-dose corticosteroids could potentially attenuate this neuroinflammatory response.

In this randomized trial, we aimed to assess the effects of an intraoperative high dose of methylprednisolone on CSF markers of neuroinflammation, brain injury, and blood-brain barrier function in patients undergoing cardiac surgery with CPB. Our hypothesis was that the previously described neuroinflammatory response to cardiac surgery is attenuated by a high dose of methylprednisolone.

## Methods

This prospective, randomized, blinded, double-armed study was approved by The Gothenburg Regional Ethics Committee (www.epn.se/goeteborg/) and the Swedish Medical Product Agency, and all patients signed an informed, written consent preoperatively. The study was registered at www.clinicaltrials.gov; identifier: NCT01755338, first posted 24 December 2012 by Mattias Danielson. This study adheres to the applicable CONSORT guidelines (see Additional file 1).

### Patients

Between January 2013 and January 2017, we enrolled 30 patients in a prospective, randomized, blinded, double-armed study. Inclusion criteria were (a) elective open aortic valve replacement surgery (SAVR) with a biological valve prosthesis due to aortic stenosis with or without concurrent coronary artery bypass grafting (CABG), (b) normal preoperative coagulation tests (i.e., partial thromboplastin time < 45 s and prothrombin time [international normalized ratio] < 1.5 and a platelet count > 80,000), (c) absence of recent (< 1 week) treatment with thrombolytic or potent anti-platelet drugs, and (d) preoperative left ventricular ejection fraction ≥ 50%. Exclusion criteria were (a) stroke with sequelae and (b) abnormal coagulation tests (see above) or thromboelastograms in the morning on the day after surgery.

### Experimental protocol

A randomized block design was used based on gender and type of surgery (SAVR or SAVR plus CABG). Patients were randomized to treatment with either methylprednisolone (Solu-Medrol^Ⓡ^, Pfizer) 15 mg/kg or placebo (0.9% sodium-chloride) administered after induction of anesthesia. A nurse, not involved in the study or care of the patients, randomized the patients to the study drugs in a 1:1 ratio, using concealed, sequentially numbered opaque envelopes. This nurse prepared the drugs and performed the blinding.

### Anesthesia and cardiopulmonary bypass

Premedication consisted of oxazepam 10 mg, oxycodone 10 mg, and half the dose of any ongoing beta-adrenergic blocker treatment orally. A catheter was placed in the radial artery before the induction of anesthesia for blood sampling and continuous monitoring of mean arterial blood pressure (MAP). Anesthesia was induced with fentanyl (5–10 μg/kg), propofol (1–1.5 mg/kg), and rocuronium (0.6 mg/kg). Before and after cardiopulmonary bypass (CPB), anesthesia was maintained with sevoflurane 0.5–2.5% in a 50% O_2_/air mixture, whereas propofol (2.5–4 mg/kg/h) was used during CPB. MAP was maintained within the range 70–80 in the pre- and post-CPB period, and a MAP within the range of 60–80 mmHg during CPB using phenylephrine and/or norepinephrine when necessary.

The CPB perfusion system consisted of a hollow fiber membrane oxygenator and a Stöckert S-5 (Sorin Group). The pump was primed with acetated Ringer’s solution and mannitol, with the target CPB flow rate of 2.4 l/min/m^2^ and the target hematocrit of 25–35%. PaCO_2_ was maintained at 40–48 mmHg and was uncorrected for body temperature. PaO_2_ was maintained at 180–260 mmHg, and SvO2 was maintained at > 65%. During CPB, the body temperature was maintained at 36 °C in all patients. During the peri- and postoperative period, the target serum glucose level was 4.2–6.3 mmol/l using a standardized insulin infusion protocol when appropriate.

### Cerebrospinal fluid (CSF) and serological markers of inflammation, brain injury, and blood-brain barrier function

A lumbar puncture for quantitative analysis of CSF biomarkers was performed the day before surgery and approximately 24 h after surgery. We used a 27-G Whitacre spinal needle (Becton Dickinson S.A. S. Agustin del Guadalix, Madrid, Spain) for median approach at L3–4 level in the sitting position. A CSF sample volume of 2.5 ml and a 4 ml blood sample were collected at both time points. Detailed information on the measurements of CSF and serological markers of inflammation, brain injury, and blood-brain barrier function are shown in Additional file 2.

### Statistical analysis

The primary end-point was the intergroup difference in postoperative IL-8. A power analysis was performed, based on our previous study [10]. To detect a 50% difference in CSF IL-8 between groups, 13 patients were needed in each group at a power of 0.80 and α of 0.05 and a standard deviation of 60 ng/L. Continuous variables were checked for normal distribution using the Shapiro-Wilk test. Within-group differences where compared using paired Student’s *t* test or Wilcoxon signed-rank test when appropriate. The comparison of the effects of surgery on changes of the various variables between the placebo and intervention groups were tested using an unpaired Student’s *t* test or Mann-Whitney *U* test. All data are presented as mean ± standard deviation (SD) or median and interquartile range (IQR) when appropriate. A probability level (*p* value) of less than 0.05 was considered to indicate statistical significance. Statistical analysis was performed using SPSS for Macintosh, version 24.

## Results

**Fig. 1:**
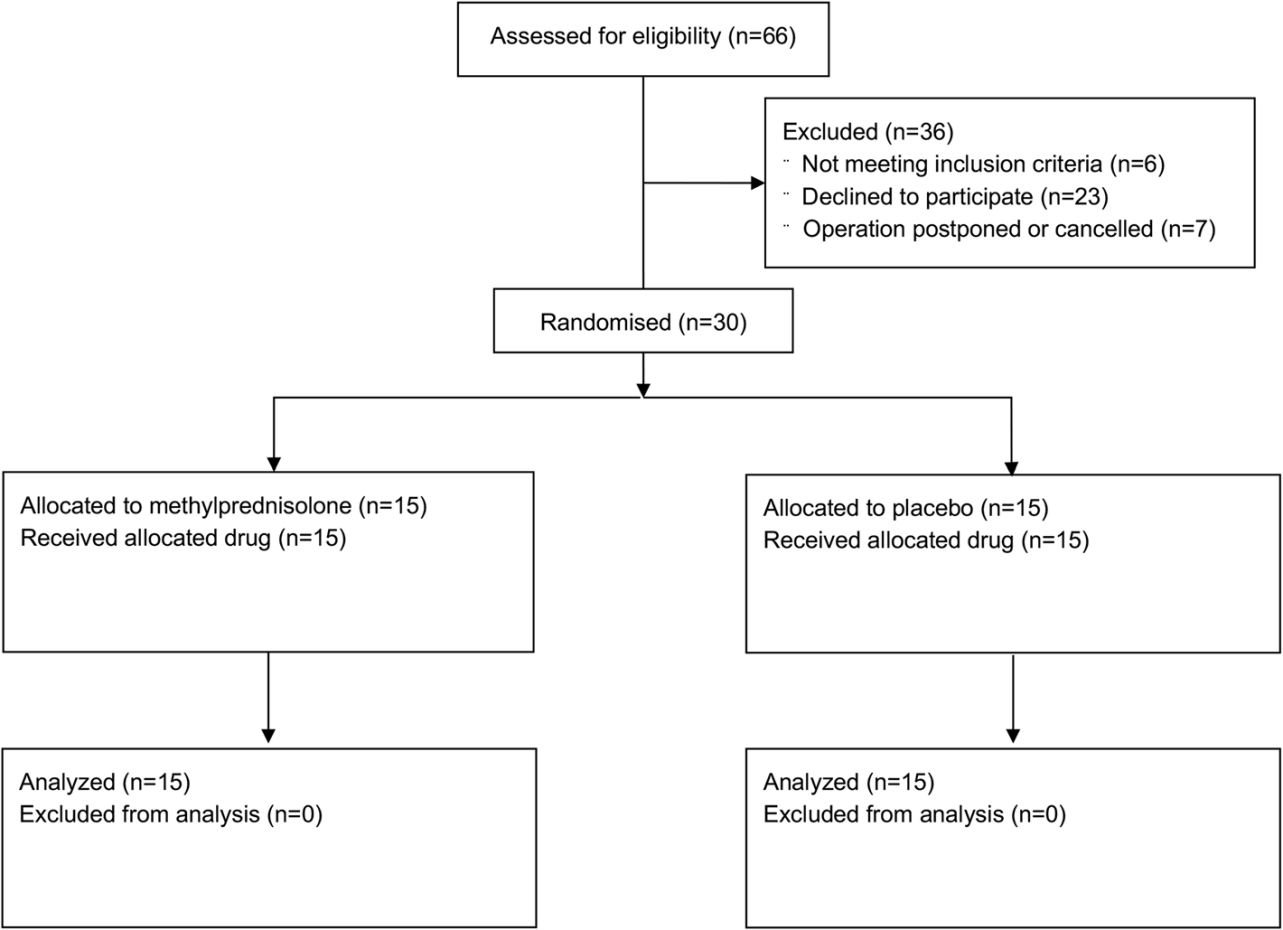
Study flow chart

The clinical trial profile is summarized in Fig. 1. The two groups were well matched with respect to age, gender, body weight, comorbidity, cardiac status, EuroSCORE II, and type of surgery (Table 1). The pre- and postoperative lumbar punctures were uneventful in all patients and in none of the patients was there a bloody tap or post-spinal headache. Intra-procedural data are presented in Table 2. There were no differences between groups with respect to durations of procedure, extracorporeal circulation, or aortic cross-clamp. Perioperative blood glucose levels did not differ between groups. There was a statistically significant increase in insulin requirement in the methylprednisolone group. Postoperatively, serum creatinine, the need for hemodynamic support, reoperation for bleeding, ICU readmission, incidence of atrial fibrillation/flutter, neurological complications, the incidence of infections, or length of ICU/hospital stay did not differ between groups.

**Table 1:** Demographic, clinical, and surgical characteristics of the groups

Characteristics	Placebo (*n* = 15)	Methylprednisolone (*n* = 15)
Demographics		
Age (years)	68.9 ± 11	70.7 ± 7.0
Male sex (*n*)	13	10
Body weight (kg)	81.1 ± 9.0	82.5 ± 15.7
Height (cm)	173 ± 7.0	176 ± 7.9
Comorbidity		
Hypertension (*n*)	9	9
Previous heart surgery (*n*)	0	0
Atrial fibrillation/flutter (*n*)	1	0
Serum creatinine (μmol/l)	89.7 ± 15	88.3 ± 20
Cardiac status		
LVEF, %	60 ± 4.2	61 ± 5.8
NYHA		
I (*n*)	7	4
II (*n*)	4	9
III (*n*)	4	2
IV (*n*)	0	0
EuroSCORE II, %	1.8 ± 1.4	1.9 ± 1.5
Type of surgery		
SAVR (*n*)	10	11
CABG + SAVR (*n*)	5	4

Values are mean ± SD. *CAGB* coronary artery bypass grafting, *LVEF* left ventricular ejection fraction, *NYHA* New York Heart association, *SAVR* surgical aortic valve repair

**Table 2:** Intra- and postoperative characteristics of the groups

Characteristics	Placebo (*n* = 15)	Methylprednisolone (*n* = 15)	*p* value
Intraoperative data			
Duration of procedure (min)	189 ± 43	204 ± 58	0.43
Duration of CPB (min)	99.4 ± 25	115 ± 31	0.14
Duration of aortic cross-clamping (min)	81.5 ± 25	91.7 ± 29	0.28
Intra-operative dose of phenylephrine (mg)	1.02 ± 1.1	1.36 ± 1.0	0.39
Intra-operative dose of norepinephrine (mg)	0.58 ± 1.3	0.56 ± 0.55	0.95
Plasma glucose levels			0.096
Start of procedure (mmol/l)	6.34 ± 1.5	5.25 ± 0.47	
During CPB (mean), (mmol/l)	6.52 ± 0.94	7.25 ± 1.0	
After CPB (mmol/l)	7.15 ± 0.97	8.50 ± 1.7	
Arrival ICU (mmol/l)	6.20 ± 0.5	7.20 ± 1.6	
Day 1 ICU (mmol/l)	6.27 ± 1.2	6.44 ± 1.2	
Insulin demand U/hour (intraoperatively + ICU)	1.14 ± 0.74	2.46 ± 1.27	0.009
Postoperative data			
Serum creatinine (μmol/l)	83.9 ± 18	94.9 ± 21	0.14
Vasopressor treatment, *n*	6	9	0.47
Inotropic treatment (*n*)	0	0	1.0
Reoperation for bleeding (*n*)	0	0	1.0
ICU readmission (*n*)	0	1	1.0
Atrial fibrillation/flutter, (*n*)	5	10	0.14
Neurological complication, (*n*)	2	1	1.0
Pneumonia, (*n*)	0	1	1.0
Wound infection, (*n*)	1	1	1.0
Other infection, (*n*)	0	2	0.48
Length of stay in ICU (hours)	24.8 ± 14	22.7 ± 12	0.65

Values are mean ± SD. *CPB* cardiopulmonary bypass, *ICU* intensive care unit. **p < 0.01

### Systemic response to cardiac surgery (Table 3)

In the control group, there was a 54-fold and 17-fold increase in serum IL-6 and IL-8, respectively. This systemic cytokine activation by cardiac surgery was clearly attenuated by methylprednisolone treatment (*p* < 0.001). Serum levels of TNF-α were not changed in the control group but decreased in the methylprednisolone group after cardiac surgery. Serum S-100B increased in both groups, but showed no difference between groups. Serum albumin decreased in both groups with no difference between groups.

**Table 3:** Systemic response to cardiac surgery

	Placebo (*n* = 15)		Methylprednisolone (*n* = 15)		Between group analysis, *p* value
	Preoperative	Postoperative	Preoperative	Postoperative	
Serum TNF-α (ng/L)#	2.45 (0.88)	2.15 (0.68)	2.36 (1.14)	1.54 (0.83)***	< 0.001
Serum IL-6 (ng/L)#	0.86 (0.82)	49.1 (48)***	0.99 (0.85)	14.6 (8.8)***	< 0.001
Serum IL-8 (ng/L)	14.2 ± 5.4	40.9 ± 23***	12.4 ± 4.8	19.2 ± 15	0.004
Serum S-100B (μg/L)#	0.060 (0.027)	0.116 (0.07)*	0.044 (0.03)	0.126 (0.06)**	0.48
Serum albumin (g/L)	43.2 ± 4.8	35.1 ± 4.5***	43.2 ± 3.3	35.9 ± 2.9***	0.65
Serum NSE (μg/L)#	14.9 (14)	27.6 (7.6)*	13.8 (8.2)	31.1 (12)*	0.27

Values are mean ± SD or median (IQR)#; not normally distributed data—non-parametric tests used. *IL-6* interleukin-6, *IL-8* interleukin-8, *NSE* neuron-specific enolase, *TNF-α* tumor necrosis factor alpha. *p < 0.05, **p < 0.01, ***p < 0.001, within-group

### Central nervous system response to cardiac surgery (Table 4)

#### Astroglial injury

There was a trend for an increase in CSF levels of S-100B in the methylprednisolone group with no differences between groups. CSF levels of GFAP were not significantly affected in any of the groups.

**Table 4:** Cerebral response to cardiac surgery

	Placebo (*n* = 15)		Methylprednisolone (*n* = 15)		Between group analysis, *p* value
	Preoperative	Postoperative	Preoperative	Postoperative	
CSF S-100B (μg/L)#	0.94 (0.26)	0.88 (0.42)	0.94 (0.36)	1.14 (0.54)*	0.47
CSF GFAP (ng/l)	643 ± 387	683 ± 388	684 ± 412	763 ± 422	0.54
CSF NSE (μg/L)#	4.55 (2.2)	4.82 (2.7)	3.60 (3.9)	6.15 (3.7)	0.36
CSF NFL (ng/L)	990 ± 442	1031 ± 442	1125 ± 583	1120 ± 527	0.43
CSF T-tau (ng/L)#	295 (233)	301 (269)*	324 (111)	288 (156)	0.085
CSF albumin (mg/L)	286 ± 80	283 ± 89	238 ± 94	255 ± 100	0.28
CSF/serum albumin ratio 95% CI	6.42 ± 1.5 (5.5–7.3)	8.0 ± 2.4** (6.7–9.3)	5.56 ± 2.3 (4.4–6.8)	7.20 ± 3.1** (5.5–8.9)	0.98
CSF TNF-α (ng/L)#	0.15 (0)	0.15 (0.08)**	0.15 (0)	0.15 (0.06)*	0.905
CSF IL-6 (ng/L)	0.833 ± 0.23	6.69 ± 4.9***	1.01 ± 0.39	2.44 ± 1.5**	0.001
CSF IL-8 (ng/L)#	40.0 (13)	130 (93)***	46.0 (15)	321 (275)***	< 0.001

Values are mean ± SD, or median (IQR)#; not normally distributed data—non-parametric tests used. *CSF* cerebrospinal fluid, *GFAP* glial fibrillary acidic protein, *IL-6* interleukin-6, *IL-8* interleukin-8, *NSE* neuron-specific enolase, *NFL* neurofilament light chain protein, *T-tau* total tau, *TNF-α* tumor necrosis factor alpha. *p < 0.05, **p < 0.01, ***p < 0.001, within-group

#### Neuronal injury

CSF levels of NSE or NFL were not affected by cardiac surgery in either of the groups. CSF levels of total tau (T-tau) decreased (− 19%) after cardiac surgery in the control but not in the methylprednisolone group.

#### Blood-brain barrier (BBB) integrity

The CSF to serum albumin ratio increased postoperatively in both the control and methylprednisolone group by 20–25%, with no difference between groups.

#### Neuroinflammation

CSF levels of TNF-α increased slightly in both the control and the methylprednisolone group after cardiac surgery with no difference between groups. There was a marked (8-fold) increase in CSF IL-6 in the control group, which was attenuated by methylprednisolone (2.4-fold increase, *p* = 0.001). Cardiac surgery induced a 3.4-fold increase in CSF-IL-8 in the control group. In the methylprednisolone group, cardiac surgery caused a 7.5-fold increase in CSF-IL-8 (*p* < 0.001).

## Discussion

The main findings were that on-pump cardiac surgery induced a systemic inflammatory response, which was accompanied by a dysfunction of the BBB and a neuroinflammatory response. Furthermore, although intra-operative preemptive treatment with a high dose of methylprednisolone, clearly attenuated the systemic inflammatory response, it did not prevent/attenuate the BBB dysfunction or the neuroinflammatory response induced by this procedure.

Cardiac surgery induced a moderate, 25% increase of the CSF to serum albumin ratio, which confirms findings from our previous study [10]. A BBB dysfunction after cardiac surgery was also demonstrated by Merino et al. who found that almost 50% of the patients had signs of BBB disruption as demonstrated by magnetic resonance imaging [9]. Increased permeability of the BBB for large molecules might explain the previously observed brain edema in patients undergoing uncomplicated on-pump cardiac surgery [8]. It has been suggested that intra-operative microembolization and the interaction between gaseous or solid cerebral emboli and the cerebral vascular endothelium might cause an activation of endothelial cells causing a secondary impaired permeability of the BBB [3, 12].

Another explanation for the increased permeability of the BBB could be the systemic inflammatory response syndrome induced by the surgical trauma. Experimental studies have shown that a peripheral sterile surgical trauma activates the innate immune system to release pro-inflammatory cytokines, increasing BBB permeability, which, in turn, will trigger inflammatory processes particularly of the hippocampal region mediated by macrophage migration through the BBB. This cerebral inflammatory response to trauma subsequently induced postoperative cognitive function in this experimental model [4, 5]. In the present study, we administered a high dose of the glucocorticoid, methylprednisolone, which induced a pronounced attenuation of the systemic cytokines TNF-α, IL-6, and IL-8, which has previously been described in several studies [13, 14]. Based on previous experimental studies, we hypothesized that such an inhibition of the systemic inflammation could prevent/attenuate the BBB dysfunction and the neuroinflammation seen after cardiac surgery. However, BBB permeability and CSF levels of TNF-α increased to a similar extent in both groups. Furthermore, there was a considerably more pronounced increase in the CSF IL-8 in the methylprednisolone group, suggesting a direct production of immune-related cells within the brain itself. On the other hand, the release of IL-6 was attenuated in the methylprednisolone group. However, changes in the CSF levels of IL-6 must be interpreted with caution, as there is a serum to CSF gradient of IL-6 both at baseline and after surgery, in contrast to what is seen for IL-8. In other words, the methylprednisolone-induced attenuation of the CSF IL-6 release could be explained by the pronounced attenuation of serum IL-6 release and consequently less passive spill-over to the CSF. Thus, the results of the present study do not support the hypothesis that the systemic inflammatory response induced by surgical trauma causes a BBB dysfunction followed by a neuroinflammatory response.

It has been suggested that increased serum levels of neuronal and glial cell injury biomarkers, such as S-100B and NSE, are associated with cerebral injury and POCD in on-pump cardiac surgery [15–17]. These studies may, however, be flawed as S-100B may originate from extracerebral sources [18] and serum levels of NSE may be derived from hemolysis during cardiopulmonary bypass [19]. The most appropriate approach to assess neuronal or glial cell injury would be to perform measurements of established biomarkers in the CSF, which is in direct contact with cerebral interstitial fluid. NSE is an enolase-isoenzyme found in neuronal and neuroendocrine tissues. Its levels in other tissues, except erythrocytes, are negligible. T-tau is released from unmyelinated cortical axons and NFL is released from subcortical myelinated axons. Data on CSF levels of neuronal and glial cell injury biomarkers before and after on-pump cardiac surgery are scarce [20, 21]. In the present study, we found no evidence of a cardiac surgery-induced neuronal injury as neither of the neuronal injury markers were elevated after surgery in the control or the methylprednisolone group. If anything, CSF levels of T-tau decreased after surgery in the control group. These findings are in line with those of Kaukinen et al. who showed that on-pump normothermic or hypothermic cardiac surgery did not cause a release of NSF into the CSF when measured days and months after the procedure [20] and results from our previous study showing that CSF levels of NSE, NFL, and T-tau were not elevated 24 h after on-pump cardiac surgery [10]. In the latter study, we found that the majority of cerebral emboli, as detected by transcranial Doppler, appeared during weaning from CPB, with a high probability of less harmful air emboli, with less cerebral flow disturbances than solid emboli. A limitation of our study is that we could not perform CSF sampling at later time points than 24 h post-surgery. Longitudinal studies following stroke suggest that the optimal time point to detect neuronal injury using CSF biomarkers is 7–10 days post-injury [22].

Data on the mechanism(s) behind the increased permeability of the BBB are not provided in the present study. Irrespective of the cause of the dysfunction of the BBB, it could not be prevented or attenuated by methylprednisolone.

**Fig. 2:**
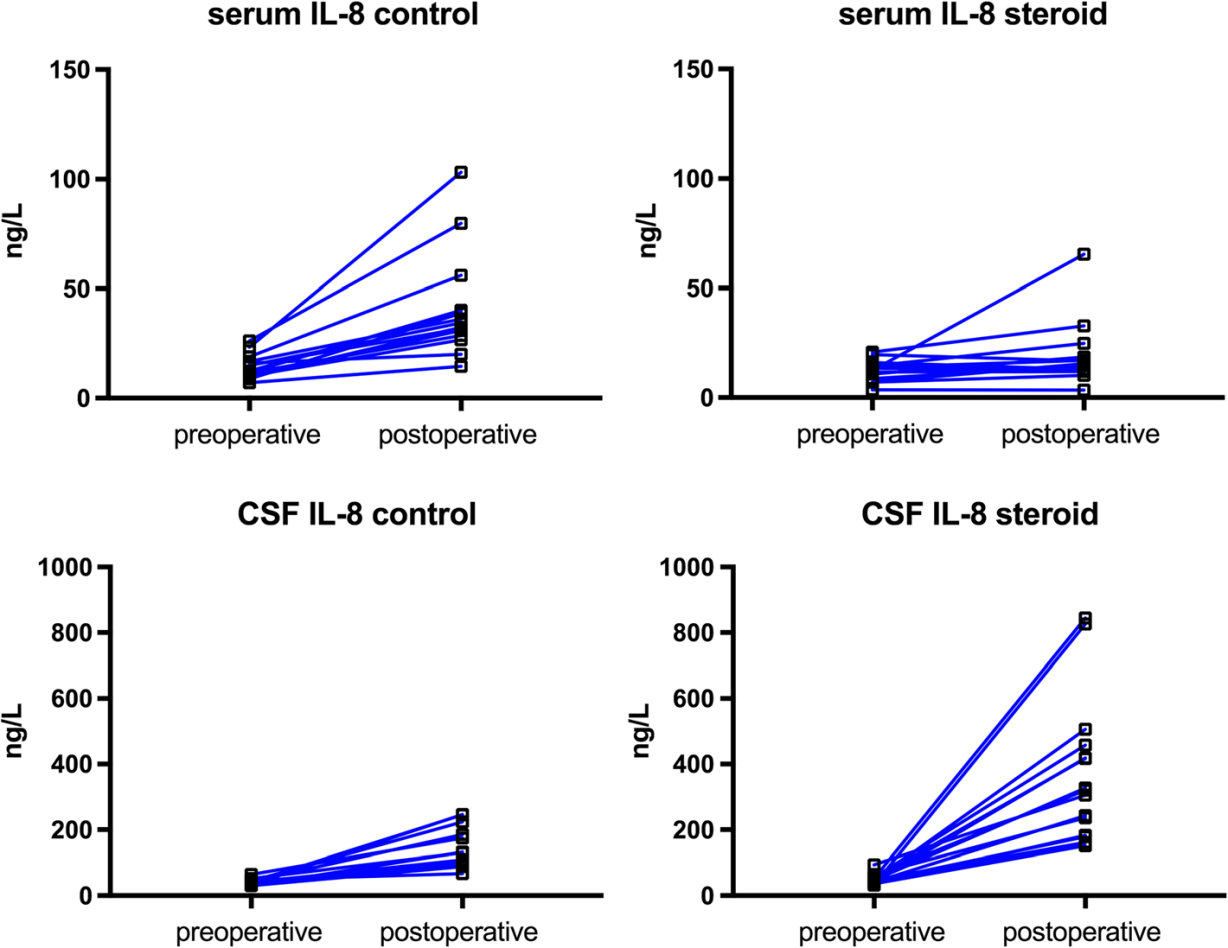
The individual data on the pre- and postoperative levels of serum and cerebrospinal fluid (CSF) levels of interleukin-8 (IL-8). The increase in serum levels of IL-8 seen after surgery was clearly attenuated by methylprednisolone (steroid), while methylprednisolone induced a pronounced accentuation of the CSF IL-8 response to cardiac surgery

IL-8 is a pro-inflammatory cytokine which attracts neutrophils as mediators of inflammation [23]. In the present study, methylprednisolone induced a more profound, 7.5-fold increase in CSF IL-8 after surgery compared to the 3.4-fold increase in the control group (Fig. 2). Data on the effects of systemic administration of a glucocorticoid on CSF levels of IL-8 are scarce. In a recent study, on the effects of intrathecal injection of methylprednisolone on postherpetic neuralgia, CSF IL-8 increased 8–9 times while the cyto/chemokines IL-1, IL-10, and TNF-α were not affected over time, compared to controls [24]. Furthermore, in a human microvascular endothelial cell line, glucocorticoids were shown to enhance ATP-induced vascular endothelial cytokine production, augmenting the release of IL-8. Thus, it seems that glucocorticoids may enhance the production of IL-8 in endothelial inflammation [25].

One limitation was that CSF levels of biomarkers were obtained only once after the surgical procedure. Thus, a more detailed evolution of the CSF biomarkers over time could not be assessed. However, it was too risky to perform more than one postoperative lumbar puncture for quantitative analysis of CSF, as anticoagulant and anti-platelet therapy was commenced on the first postoperative day. Furthermore, we did not measure anti-inflammatory cytokines, which could have provided information on the effects of cardiac surgery on the balance between pro- and anti-inflammatory mediators in the CSF and the potential effect of preemptive methylprednisolone on this balance.

## Conclusions

In conclusion, we showed that preventive treatment with high-dose methylprednisolone attenuated the systemic inflammatory response to cardiac surgery with CPB, but this treatment did not prevent or attenuate the increase in BBB permeability or the neuroinflammatory response seen after this procedure.

## Additional files

Additional file 1: CONSORT 2010 Checklist for reporting of a randomized trial. (DOC 218 kb)

Additional file 2: Measurements of cerebrospinal fluid (CSF) and serological markers of inflammation, brain injury and blood-brain barrier function. (DOCX 14 kb)

## Abbreviations

BBB: Blood-brain barrier
CABG: Coronary artery bypass grafting
CPB: Cardiopulmonary bypass
CSF: Cerebrospinal fluid
GFAP: Glial fibrillary acidic protein
ICU: Intensive care unit
IL-6: Interleukin-6
IL-8: Interleukin-8
IQR: Interquartile range
LVEF: Left ventricular ejection fraction
MAP: Mean arterial blood pressure
NFL: Neurofilament light chain protein
NSE: Neuron-specific enolase
NYHA: New York Heart Association
POCD: Postoperative cognitive dysfunction
SAVR: Surgical aortic valve replacement
SD: Standard deviation
TNF-α: Tumor necrosis factor alpha
T-tau: Total tau
