# Supplementary material for: Effects of methylprednisolone on blood-brain barrier and cerebral inflammation in cardiac surgery—a randomized trial
Source: J Neuroinflammation. 2018 Sep 27;15:283. doi: 10.1186/s12974-018-1318-y (PMC6158839; doi:10.1186/s12974-018-1318-y)
Supplement: Supplementary file 2 — Measurements of cerebrospinal fluid (CSF) and serological markers of inflammation, brain injury and blood-brain barrier function. (DOCX 14 kb) [file 12974_2018_1318_MOESM2_ESM.docx]

**Measurements of cerebrospinal fluid (CSF) and serological markers of inflammation, brain injury and blood-brain barrier function**

Serum and CSF levels of S-100B were determined by an electrochemo-luminescence immunoassay using the Modular system and the S-100B reagent kit (Roche Diagnostics, Basel, Switzerland). Glial fibrillary acidic protein (GFAP) concentrations in serum and CSF were measured using a previously described ELISA method ([15](#_ENREF_15)). Both S-100B and GFAP are biomarkers of astroglial injury. CSF and serum levels of NSE were measured using an immunofluorescent assay with time-resolved amplified cryptate emission (TRACE) technology (Kryptor-NSE; BRAHMS, Hennigsdorf, Germany). CSF total tau (T-tau) concentration was determined using a sandwich enzyme-linked immunosorbent assay (ELISA) (INNOTEST hTAU-Ag; Fujirebio, Gent, Belgium) specifically constructed to measure all tau isoforms irrespective of phosphorylation status. CSF neurofilament light (NFL) concentration was determined using a commercial assay as described by the manufacturer (UmanDiagnostics AB, Umeå, Sweden).

CSF and serum levels of TNF-α, IL-6 and IL-8 were determined using the Human Proinflammatory II 4-Plex Assay, Ultra-Sensitive Kit, with electrochemiluminescent detection according to the instructions from the manufacturer (Meso Scale Discovery®, Rockville, MD, USA). IL-6 and IL-8 were readily measurable in all CSF samples. Intra-assay coefficients of variation were below 10% for all analyses. Albumin levels in CSF (mg/L) and serum (g/L) were measured by immunonephelometry on an Immage immunochemistry system (Beckman Coulter Inc, Fullerton, Calif.), and the CSF/serum albumin ratio was calculated both pre-and postoperatively**.**
